# Multi-parametric MRI-based machine learning model for prediction of pathological grade of renal injury in a rat kidney cold ischemia-reperfusion injury model

**DOI:** 10.1186/s12880-024-01320-6

**Published:** 2024-07-26

**Authors:** Lihua Chen, Yan Ren, Yizhong Yuan, Jipan Xu, Baole Wen, Shuangshuang Xie, Jinxia Zhu, Wenshuo Li, Xiaoli Gong, Wen Shen

**Affiliations:** 1 Department of Radiology, Tianjin First Central Hospital, Tianjin Institute of Imaging Medicine, No. 24 Fu Kang Road, Nan Kai District, Tianjin, 300192 China; 2 Department of Radiology, Tianjin Medical University General Hospital, Tianjin, 300052 China; 3 College of Medicine, Nankai University, Tianjin, 300350 China; 4 MR Collaborations, Siemens Healthcare China, Beijing, 100102 China; 5 College of Computer Science, Nankai University, Tianjin, 300350 China

**Keywords:** Machine learning, Renal injury, Cold ischemia-reperfusion injury, Multi-parametric MRI, Pathological grade

## Abstract

**Background:**

Renal cold ischemia-reperfusion injury (CIRI), a pathological process during kidney transplantation, may result in delayed graft function and negatively impact graft survival and function. There is a lack of an accurate and non-invasive tool for evaluating the degree of CIRI. Multi-parametric MRI has been widely used to detect and evaluate kidney injury. The machine learning algorithms introduced the opportunity to combine biomarkers from different MRI metrics into a single classifier.

**Objective:**

To evaluate the performance of multi-parametric magnetic resonance imaging for grading renal injury in a rat model of renal cold ischemia-reperfusion injury using a machine learning approach.

**Methods:**

Eighty male SD rats were selected to establish a renal cold ischemia -reperfusion model, and all performed multiparametric MRI scans (DWI, IVIM, DKI, BOLD, T1mapping and ASL), followed by pathological analysis. A total of 25 parameters of renal cortex and medulla were analyzed as features. The pathology scores were divided into 3 groups using K-means clustering method. Lasso regression was applied for the initial selecting of features. The optimal features and the best techniques for pathological grading were obtained. Multiple classifiers were used to construct models to evaluate the predictive value for pathology grading.

**Results:**

All rats were categorized into mild, moderate, and severe injury group according the pathologic scores. The 8 features that correlated better with the pathologic classification were medullary and cortical Dp, cortical T2*, cortical Fp, medullary T2*, ∆T1, cortical RBF, medullary T1. The accuracy(0.83, 0.850, 0.81, respectively) and AUC (0.95, 0.93, 0.90, respectively) for pathologic classification of the logistic regression, SVM, and RF are significantly higher than other classifiers. For the logistic model and combining logistic, RF and SVM model of different techniques for pathology grading, the stable and perform are both well. Based on logistic regression, IVIM has the highest AUC (0.93) for pathological grading, followed by BOLD(0.90).

**Conclusion:**

The multi-parametric MRI-based machine learning model could be valuable for noninvasive assessment of the degree of renal injury.

**Supplementary Information:**

The online version contains supplementary material available at 10.1186/s12880-024-01320-6.

## Introduction

Renal cold ischemia-reperfusion injury (CIRI), a pathological process during kidney transplantation, may result in delayed graft function and negatively impact graft survival and function [1]. Early detection of allograft injury, before serum creatinine concentration (SCr) or blood urea nitrogen(BUN) rising which are widely used to evaluate renal injury, may improve long term allograft survival. Several biomarks including kidney injury molecule-1(KIM-1), cystatin C, neutrophil gelatinase-associated lipocalin (NGAL), may be sensitive to detect kidney damage, but have not be used in the clinical setting [2, 3]. The gold standard is protocol biopsy to monitor allograft injury currently, which is invasive with the risk of allograft bleeding and sampling error [4].

The potential of multi-parametric quantitative magnetic resonance imaging (MRI) has been extensively discussed as a diagnostic tool in renal function. Functional MRI techniques without the use of exogenous contrast agents, can indirectly reflect renal injury reduced by acute kidney injury, chronic kidney disease, renal allograft dysfunction, and so on, by quantitatively assessing blood flow with arterial spin labeling(ASL) [5], blood oxygen with blood oxygen level-dependent (BOLD) [6, 7], water content with longitudinal relaxation time (T1) mapping and water molecule diffusion with diffusion weighted imaging (DWI) [8, 9]. Intravoxel incoherent motion (IVIM), which applies a bi-exponential model using multiple b-values, can incorporate the pure diffusion of water molecules as well as the pseudo-diffusion component formed by microcirculation or perfusion, microvascular dynamics [10, 11]. Due to the obvious directionality of the medullary renal tubules and collecting ducts arrangement, the water molecules motion in kidney is non-Gaussian distribution, especially in medulla, making diffusion kurtosis imaging (DKI) a potentially useful technique to assess complexity of the renal microstructure [12]. Renal cortical and medullary T1 values measured by T1 mapping may increase and renal blood flow (RBF) values may decrease due to inflammation, edema, thrombus, or renal structural damage [5, 8].

After kidney injury, a series of microstructural changes occur in tissue blood perfusion level, blood oxygen metabolism and water molecule diffusion, which can be detected by MRI. We hypothesize that the combination of multiple parameters can further quantitatively assess the extent of kidney injury. The machine learning algorithms introduced the opportunity to combine biomarkers from different MRI metrics into a single classifier. The integration of multi-quantitative parameters into a useful diagnostic classifier may improve diagnostic accuracy, which has been proved by several researches [13–15].

In the present study, a retrospective data analysis with parameter values obtained from multiple MRI techniques (ASL, conventional DWI, IVIM, DKI, BOLD, T1 mapping) was conducted in a rat kidney cold ischemia reperfusion injury model. Thus, this study aimed to evaluate the performance of multiple biological parameters derived from multi-parametric MRI and various machine learning classifiers, for the degree of renal injury preoperatively, when pathology as the gold standard.

## Methods

### Study design and animals

This study received approval from the Ethics Committee of Nankai University. All researchers strictly adhered to the animal ethical guidelines of our institutional animal care and use committee and the ARRIVE (Animal Research: Reporting In Vivo Experiments) guidelines during performing experiments, and made effort to minimize the number of animals used and their suffering. 80 healthy male Sprague Dawley rats weighing between 200 g and 250 g at 8–10 weeks of age, were randomly divided into four groups: sham operation group, 1 h, 2 h, and 4 h cold ischemia group (*n* = 20 rats in each group).

The establish of renal cold ischemia-reperfusion injury rat model were referenced the previous research [16] and followed by the steps. In the cold ischemia groups, the blood vessels of the left renal pedicle were clipped. The left kidney was injected with perfusion fluid until it became pale, and then ice chips were placed around the kidney for 1 h, 2 h, and 4 h, respectively. And then the right kidney was removed after the chipping was removed. In the sham operation group, only the right kidney was removed. Then 5 rats at 1 h, day 1, day 2 and day 5 after operation were randomly selected for MRI for each group. Finally, the left kidney was taken and stored in 4% paraformaldehyde solution for morphological score. During the experiment, the number of rats was increased at any time, when the image quality did not meet the measurement requirements.

### MRI imaging

All MRI examinations were performed on a 3T MR scanner (MAGNETOM Prisma, Siemens Healthcare, Erlangen, Germany) with an 8-channel experimental animal coil (Shanghai Chenguang Medical Technology Co., Ltd., Shanghai, China). The rats were anesthetized using 5% isoflurane inhalation with a flow rate of 1 L/min before MRI scan and using 2% isoflurane maintenance with a flow rate of 0.5 L/min during scanning. The MR protocols contained a T2-weighted turbo spin-echo (TSE) sequence and ASL, Multi-b-value DWI, BOLD and T1 mapping under free breathing conditions. During the scanning process, the scanning center of each sequence should be as consistent as possible, including T2 conventional sequences.

The coronal T2-weighted TSE sequence was performed for morphological evaluation of the kidneys using parameters as follows: repetition time(TR): 4120ms, echo time (TE): 100 ms, field of view (FOV): 100 mm × 75 mm, slice thickness: 1.5 mm, matrix: 192 × 192, reconstructed voxel size: 0.3 × 0.3 × 1.5 mm^3^, acquisition time of 4 min and 13 s.

ASL MRI was performed in the axial plane with the following parameters: TR, 6000ms, TE, 49.8ms, inversion time (TI), 1200ms, FOV, 153 mm × 153 mm, slice thickness, 3 mm, reconstructed voxel size, 1.2 × 1.2 × 3.0 mm^3^, acquisition time, 7 min and 5 s.

Multi-b-value DWI (b-values of 0, 10, 20, 30, 50, 100, 200, 300, 500, 800, 1000, 1500, and 2000 s/mm^2^) was performed on three gradient directions and in the coronal plane with the integrated shiming (iShim) technique. The parameters were as follows: TR/TE, 1500.0ms/60.0ms, 14 slices with thickness of 5 mm, FOV, 300 × 300 mm^2^, matrix, 120 × 98 mm, reconstructed voxel size, 0.6 × 0.6 × 3.0 mm^3^, acquisition time, 10 min and 22 s.

BOLD MRI was performed in the coronal T2* map using a multiple gradient echo sequence with the following parameters: TR, 2500 ms, 6 TEs, 3.22, 5.83, 8.42, 11.01, 13.63, 16.22 ms, FOV, 85 mm× 62 mm, slice thickness, 3.0 mm, matrix, 160 × 160, reconstructed voxel size, 0.5 × 0.5 × 3.0 mm^3^, acquisition time of 3 min and 57 s.

T1 mapping MRI was performed in the coronal plane with the following parameters: TR, 6.79 ms, TE, 2.85 ms, flip angles, 2° and 10°, FOV, 80 mm × 60 mm, slice thickness, 2.0 mm, matrix, 140 × 100, reconstructed voxel size, 0.3 × 0.3 × 2.0 mm^3^, acquisition time, 5 min and 41 s.

### Imaging processing

All the raw images were transferred to the Siemens Syngo.via Frontier post-processing workstation (Siemens Healthcare, Erlangen, Germany), which automatically generates RBF maps, T2*, T1 mapping maps and pseudo-color maps.

The iShim multi-b-value DWI images were processed and analyzed using prototypic software syngo.via Research Frontier (MR Body Diffusion Toolbox version 1.6.0 and MR Multiparametric Analysis version 1.2.1, Siemens Healthcare, Erlangen, Germany). All b-value images were selected to generate apparent diffusion coefficient (ADC) maps from the mono-exponential model according to the equation [17]: S(b)/S(0) = exp(-b×ADC). Ten b-values (0, 10, 20, 30, 50, 100, 200, 300, 500, and 800 s/mm^2^) were selected to generate parametric maps from the bi-exponential fitting according to the equation [18]: S(b)/S(0) =(1 − Fp)×exp(− b×D) + f×exp (− b×Dp). S(b) represents the signal intensity in the presence of diffusion sensitization and S(0) represents the signal intensity in the absence of diffusion sensitization. D (×10^− 3^ mm^2^/s) represents predominantly pure molecular diffusion, when Dp (×10^− 3^mm^2^/s) is the pseudodiffusion coefficient dominated by the much faster microcirculation or perfusion; and Fp(%) represents the perfusion fraction. Five b-values (0, 500, 1000, 1500, and 2000 s/mm^2^) were selected to generate parametric maps from the kurtosis model according to the equation [19]: S(b)/S(0) = exp(− b×D + 1/6×b^2^×Md^2^×Mk), including Mk is mean kurtosis, and Md is mean diffusivity.

### MR quantitative measurement and analysis

**Fig. 1:**
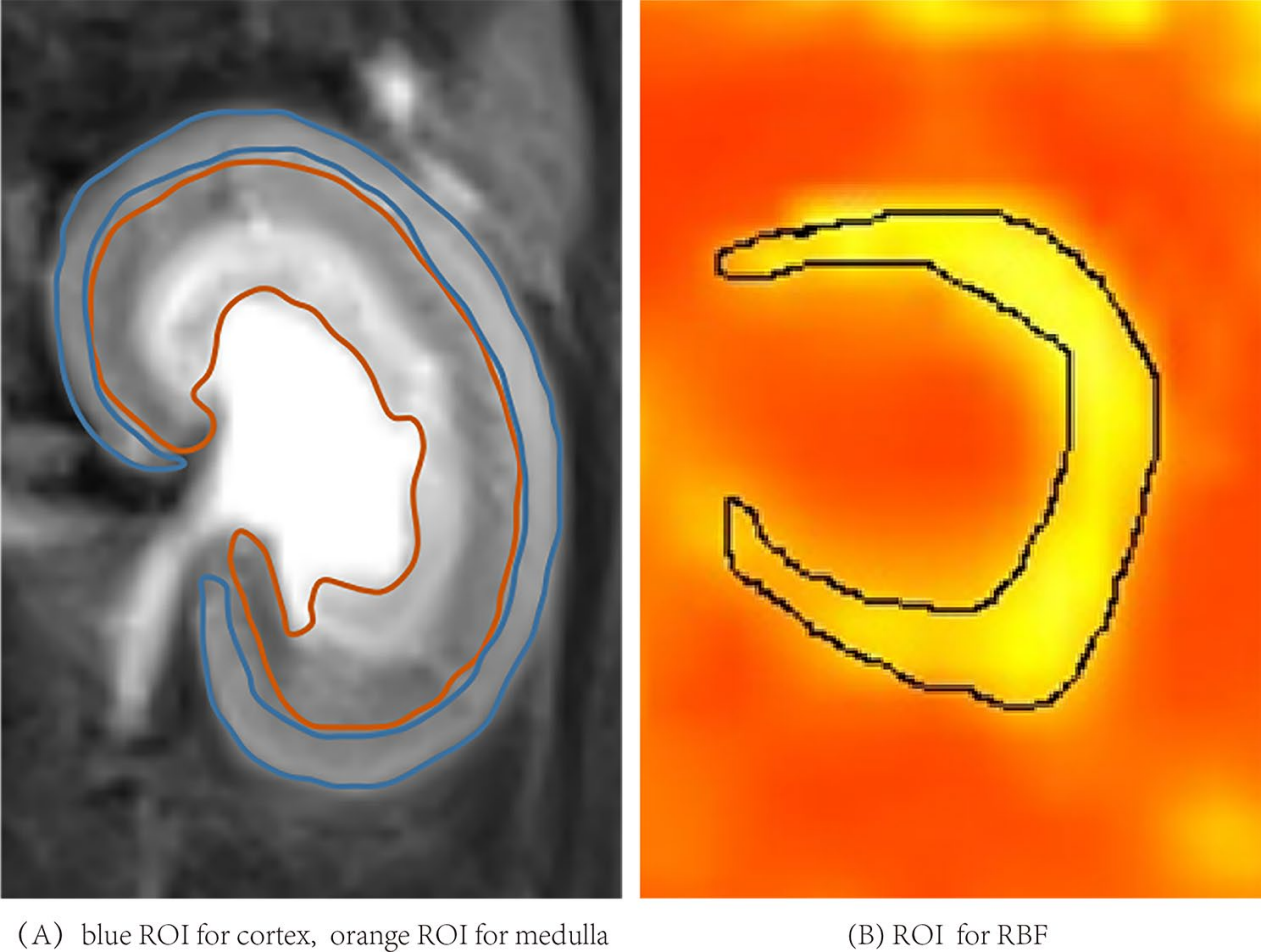
The region of interest (ROI)

All images were measured and analyzed by two expert radiologists (L.C. and Y.R.) with 15 and 6 years of experience in diagnostic abdominal MRI imaging, respectively, who were blinded to the data. IVIM-derived parameters (D, Dp, and Fp), DKI-derived parameters (Md and Mk), ADC, T2* and T1 values of cortex and medulla, and cortical RBF value were measured. On the largest section of kidney on each parameter map, the renal cortex and medulla (including the outer stripe and the inner stripe of the outer medulla) region were outlined manually with the T2-weighted images as the reference, avoiding inner medulla, artifacts and perinephric effusion (Fig. 1-A). The region of interest (ROI) on the RBF map includes the cortex and the outer stripe of the outer medulla (Fig. 1-B).

The categorical features included cortical and medullary ADC, D, Dp, Fp, Mk, Md, T2*, T1 values and difference between cortex and medulla of each parameter (∆ADC, ∆D, ∆Dp, ∆Fp, ∆T2*, ∆Mk, ∆Md, ∆T1), cortical RBF values. A total of 25 features were analyzed to predict the degree of renal injury preoperatively.

Feature Selection: The least absolute shrinkage and selection operator (LASSO) regression was used for feature selection. To determine the optimal regression parameters (λ), fivefold cross-validation was used. In each step of the cross-validation, 1 fold of the dataset is treated as the test set and the remaining 4 folds are used for training. The λ-value was selected at the minimum mean squared errors (MSE) on the average of MSE curve of fivefold cross-validation. Then, the optimal features were extracted based on the optimal λ-value with *P* value < 0.05 both for the positive and negative *t*-test and the analysis of variance (ANOVA) of the features.

**Fig. 2:**
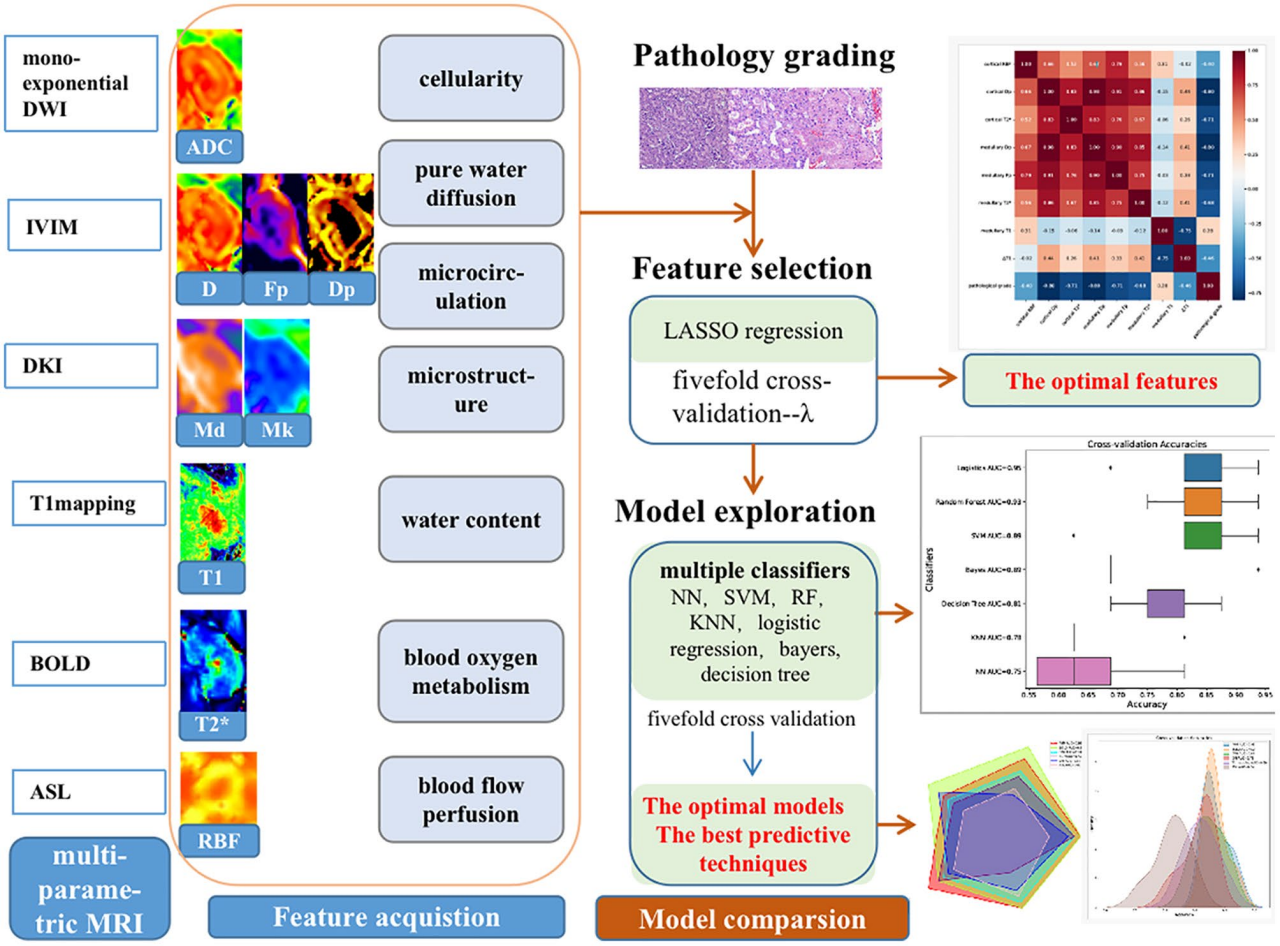
The workflow of grading renal injury with multiple MRI biological markers and machine learning classifiers

After the optimal features were obtained, multiple classifiers including neural network (NN), support vector machine (SVM), random forest (RF), multinomial logistic regression (logistics), bayers, decision tree, and K Nearest Neighbors (KNN), were used to perform a 5-fold cross-validation. Compared the average accuracy, area under curve(AUC) with One-vs-Rest(OVR) multiclass classification approach, F1-score of these models, which were used as the evaluation indicators. The optimal model based on the features to predict the degree of renal injury preoperatively was obtained. Finally, the grading efficacy of the optimal model for different MRI techniques was compared, which included DWI (cortical and medullary ADC, ∆ADC), IVIM (cortical and medullary D, Dp, Fp, ∆D, ∆Dp, ∆Fp), DKI (cortical and medullary Mk, Md, ∆Mk, ∆Md), BOLD (cortical and medullary T2*, ∆T2*), T1mapping (cortical and medullary T1, ∆T1) and ASL (cortical RBF). AUC value was used as the evaluation indicator. And the best predictive technique and model for grading the renal injury were derived. The workflow of study is shown in Fig. 2.

### Histopathological analysis

The pathomorphological characteristics of renal tissues were observed by hematoxylin and eosin (HE) staining. The semi-quantitative scoring of renal tubule injury was evaluated with reference to the Paller’s standard [20]. The pathological scoring criteria were detailed in supplementary material (Supplementary Table S1). Five random fields were chosen at high-power field (× 400), and 10 renal tubules were selected for scoring in each field of view. Higher scores indicated more severe injury. The values of a total of 50 tubules to be scored were averaged.

### Statistical analysis

Statistical analysis was performed with SPSS 26.0 software (SPSS Inc., Chicago, IL, USA) and R language software (version 4.1.2). The histopathological scores were divided into three groups corresponding to mild, moderate and severe using *K*-means clustering method. Spearman correlations were used to analyze the correlations between the parameters generated from multi-parameter MRI and histopathological score. The continuous variables were described as mean and standard deviation (SD). The features for grading the pathological injury were compared by the positive and negative *t*-test and the ANOVA.

Intraclass correlation efficient (ICC) with 95% confidence interval (CI) was used to assess inter-observer reliability. ICCs range from 0 to 1 are commonly classified as follows: ICC ≤ 0.75 means poor agreement; ICC 0.75–0.9 means moderate agreement; and ICC > 0.90 means high agreement. Results with *P* values less than 0.05 were considered statistically significant.

## Results

### Interobserver agreement

No significant differences in all MRI parameters were observed between two observers (*P* > 0.05). All ICCs between two observers demonstrated good to excellent range (ICC = 0.735–0.992, CI = 0.616–0.995, all *P* > 0.05). The ICC and 95% confidence interval were detailed in supplementary material (Supplementary Table S2). And the averages of the parameters were used for the following analysis.

### Histopathologic score grading of Renal Injury

**Fig. 3:**
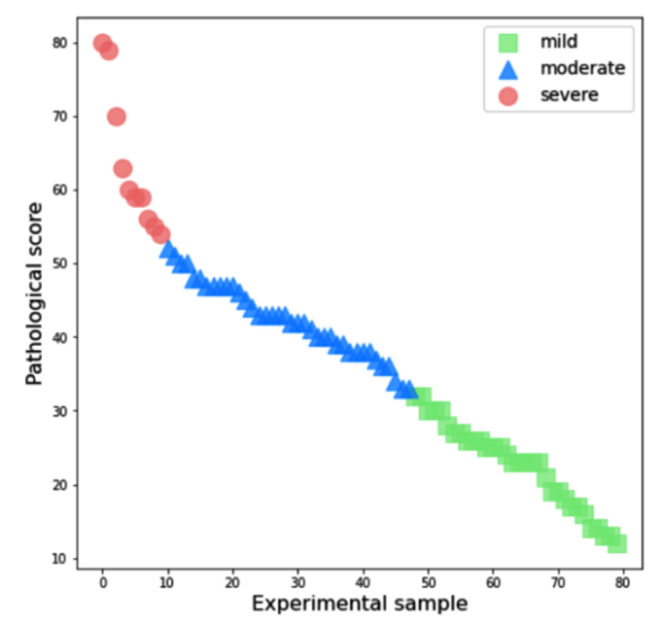
Cluster analysis for grading kidney injury histopathological score based on K means

Clustering analysis (k = 3) of the histopathological scores of renal injury based on the K-means method showed that pathological scores less than 33 were mild, more than 52 were severe, and 33–52 were for moderate impairment (Fig. 3).

### Feature selection

**Fig. 4:**
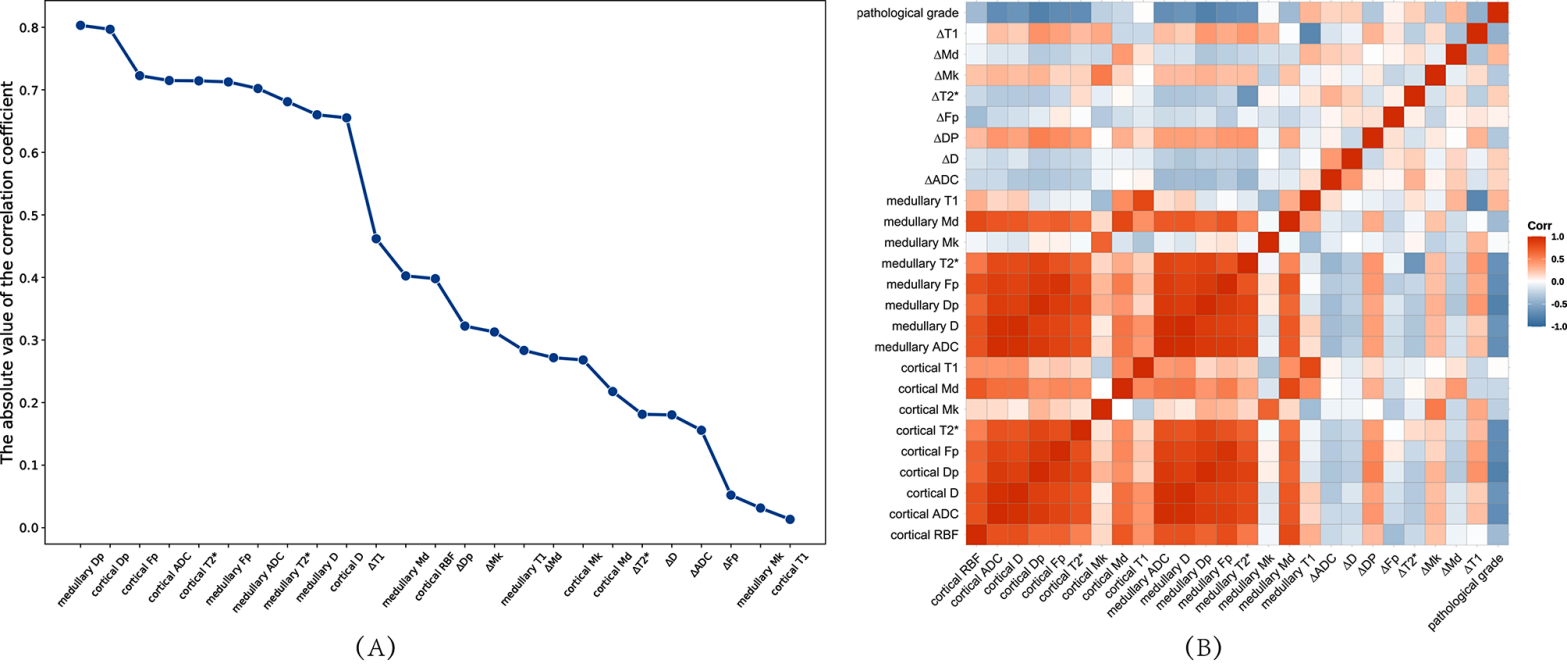
Line graph (**A**) and heat maps (**B**) of the correlation between MRI parameters and pathology grading

The correlation between the values of each MRI parameter and the pathological grading is shown in Fig. 4.

**Fig. 5:**
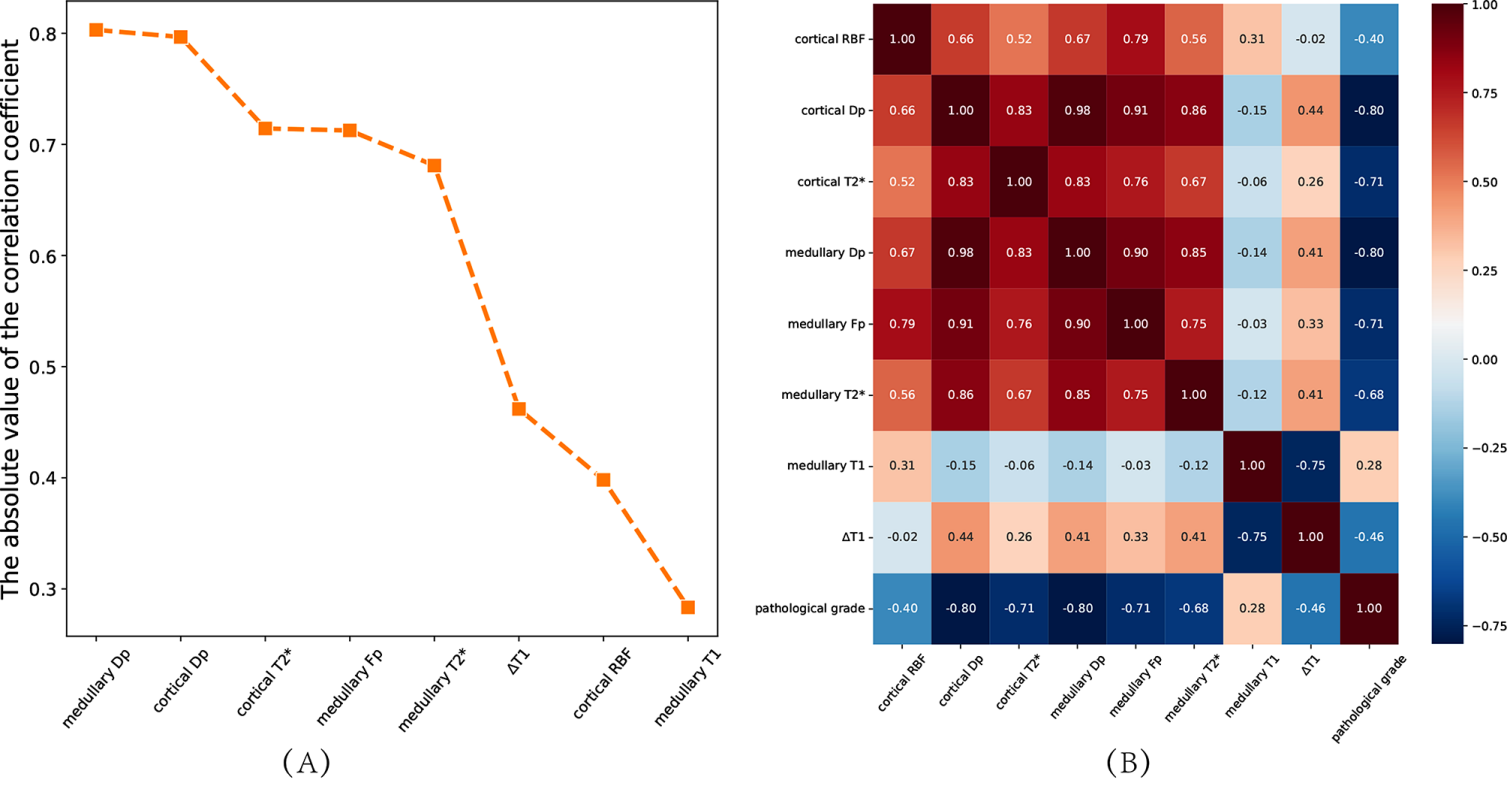
Line graph (**A**) and heat maps (**B**) of the correlation between the optimal characteristics and pathology grading

After LASSO regression analysis, the optimal λ-value was selected by the five-fold cross-validation, λ = 0.049417133623833. Then, nine relevant features were extracted based on the optimal λ-value. Eight features (medullary Dp, cortical Dp, cortical T2*, cortical Fp, medullary T2*, ∆T1, cortical RBF, medullary T1) with *P* value < 0.05 both for the *t*-test and the ANOVA, were filtered out finally. The correlations between 8 features and pathological grading and correlation between features were shown in Fig. 5.

### Predictive model construction for grading renal injury

**Fig. 6:**
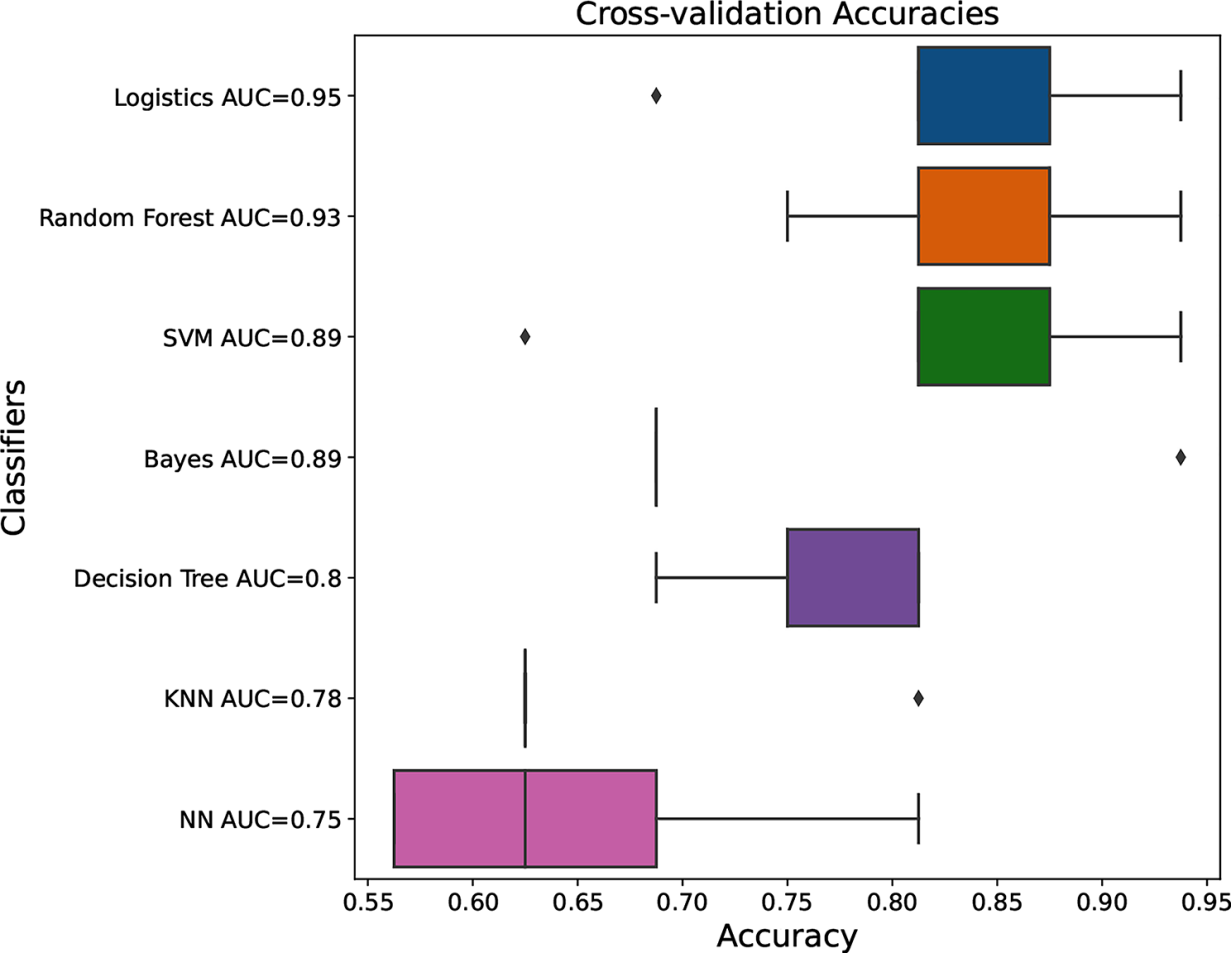
Box plots of the accuracy of different models constructed based on 8 optimal features. Logistics, multinomial logistic regression, SVM, support vector machine, KNN, K Nearest Neighbors, NN, neural network, AUC, area under curve

**Fig. 7:**
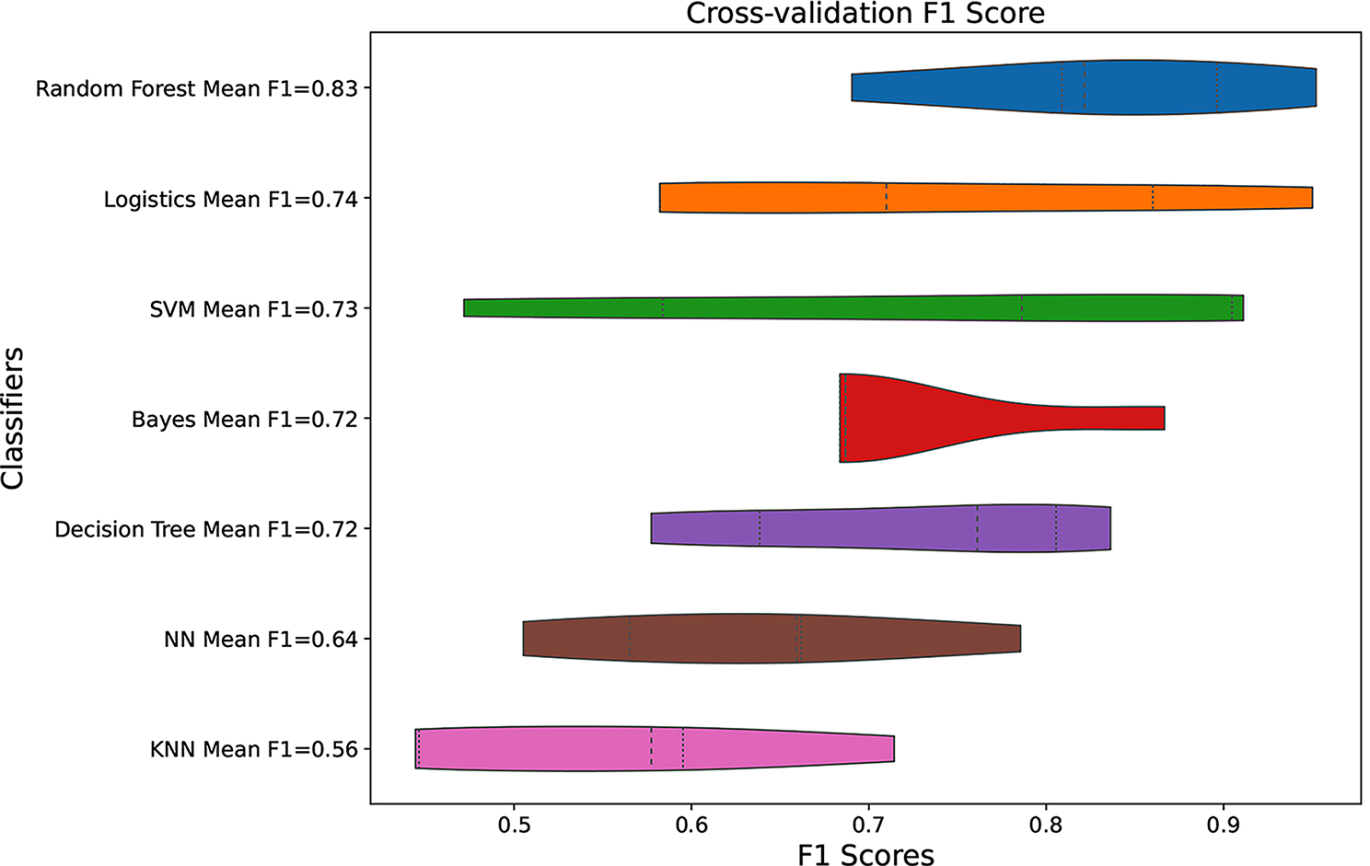
Violin plots of F1 scores for different models constructed based on 8 optimal features. Logistics, multinomial logistic regression, SVM, support vector machine, NN, neural network, KNN, K Nearest Neighbors

Based on the 8 optimal features selected, multiple classifiers including neural network (NN), support vector machine (SVM), random forest (RF), multinomial logistic regression(logistics), bayers, decision tree, and K Nearest Neighbors (KNN) were used to construct the best predictive model for pathology grading. The accuracy of logistics, RF and SVM were significantly higher than others (*P* < 0.05), and the results were shown in Fig. 6. There was no statistical difference of the accuracy among logistics, RF and SVM (*P* > 0.05), which were used subsequent analysis. The logistics model had a higher AUC value than RF, and F1 score of RF was higher than logistics (Fig. 7). The accuracy, AUC and F1 score of each model were shown in supplementary material (Supplementary Table S3). Three rats were randomly selected for internal validation of the predictive model, and the results were shown in Table 1.

**Table 1:** The internal validation of predictive model for grading renal injury

	Probability of grade 0	Probability of grade 1	Probability of grade 2
Rat 1	2.37646186e-07	2.10823979e-01	**7.89175783e-01**
Rat 2	**9.99911137e-01**	8.84122199e-05	4.50636708e-07
Rat 3	5.63593840e-05	**9.99156235e-01**	7.87405254e-04

Grade 0: the renal injury was mild, grade 1: the kidney was moderate impairment, grade 2: the renal injury was severe

### The best predictive techniques and models for grading

**Fig. 8:**
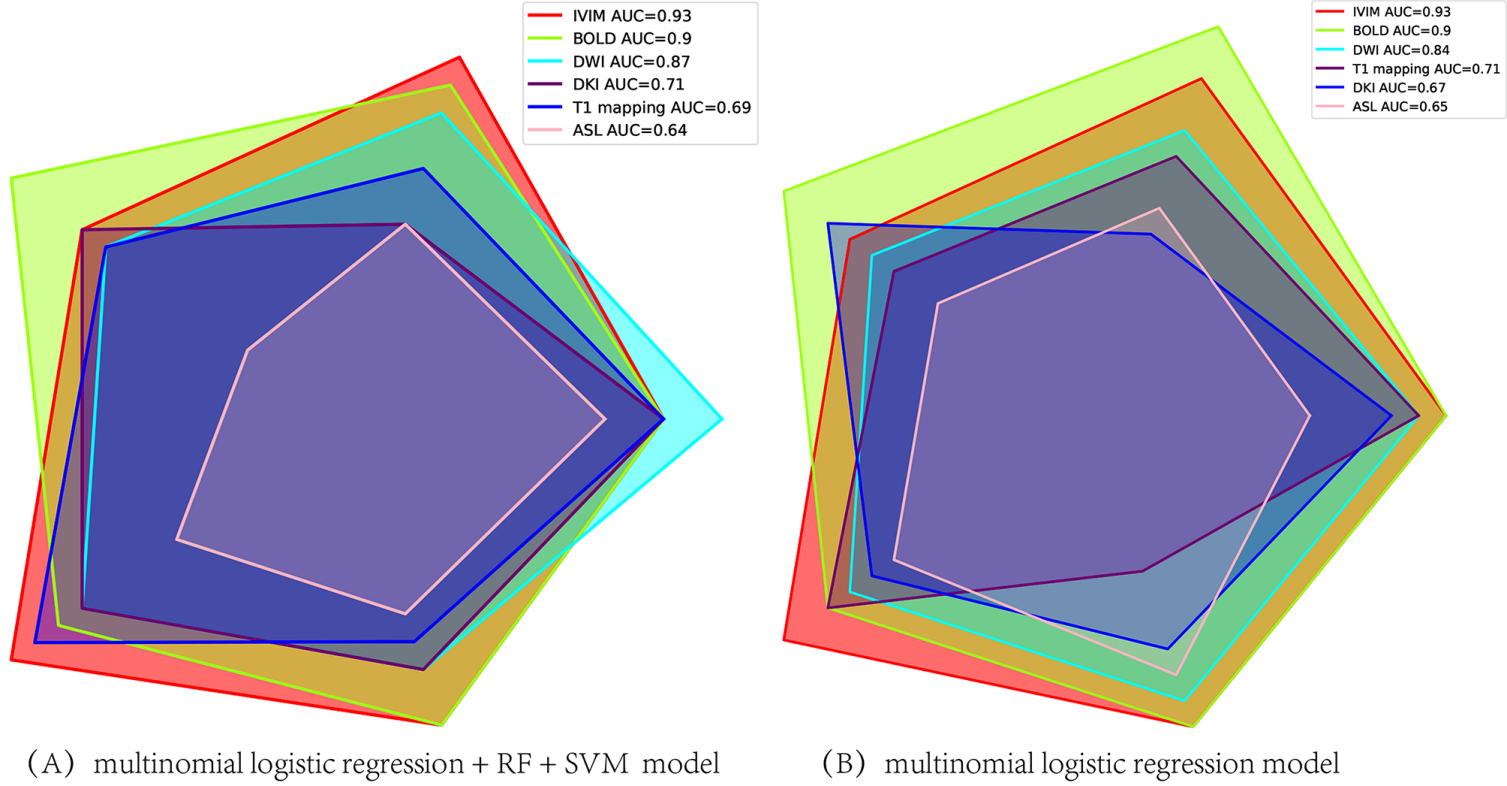
Radar plot of accuracy of different techniques for pathology grading by machine learning

**Fig. 9:**
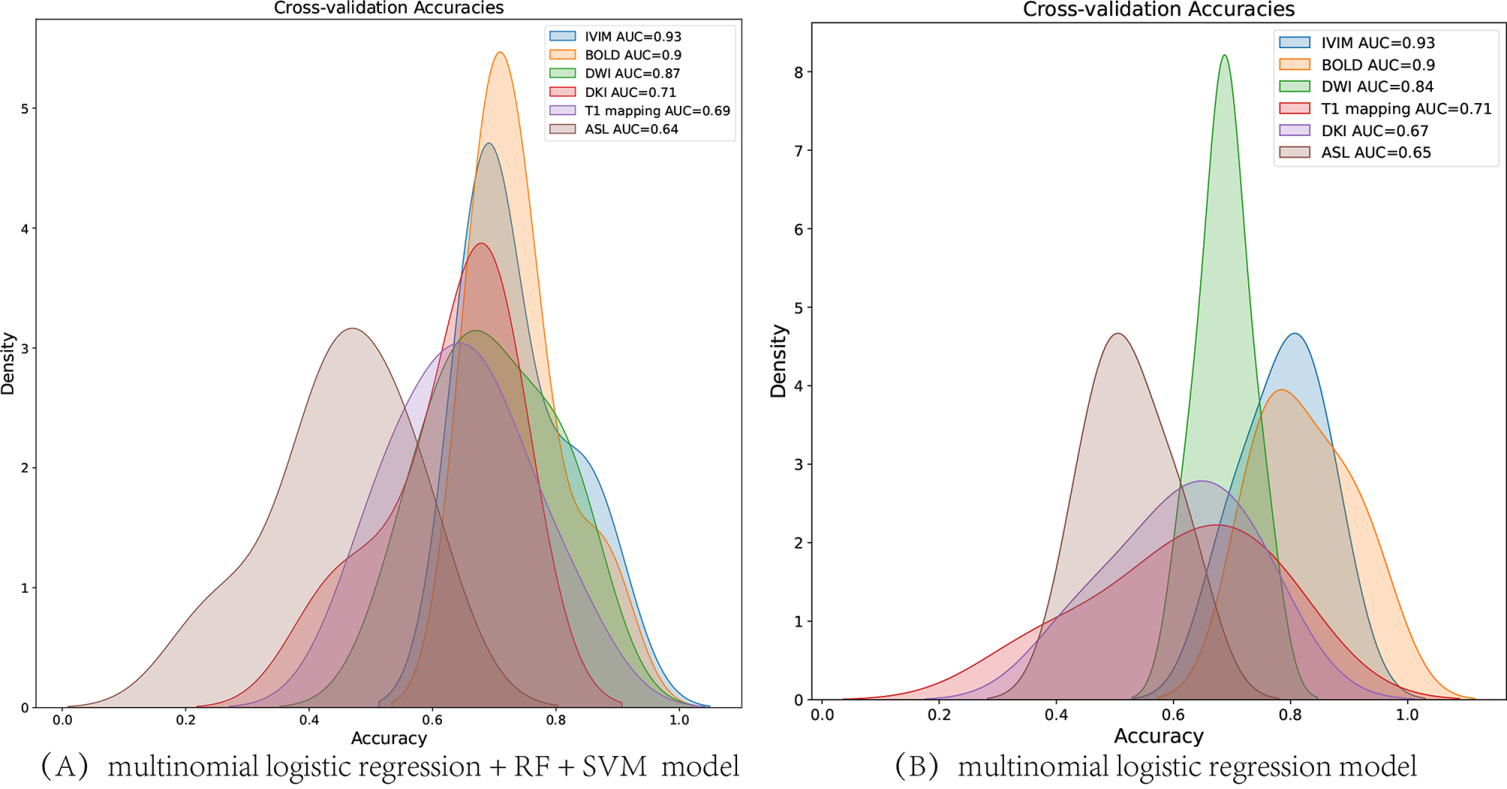
Density plots of the distribution of the accuracy by different techniques for pathology grading

We compared the grading efficacy of combined model (logistics + RF + SVM) and logistics models for different techniques. Radar plot and density plots reflected there were good and stable model performance both in logistics + RF + SVM and logistics model (Figs. 8 and 9). IVIM technique had the highest AUC for pathology grading, followed by BOLD MRI.

## Discussion

The present study had two important findings. First, we established a simple classifier model to predict the degree of kidney injury quantitatively based on the optimal features (medullary Dp, cortical Dp, cortical T2*, cortical Fp, medullary T2*, ∆T1, cortical RBF, medullary T1), while pathology as the gold standard. Second, we found that IVIM and BOLD technique had the higher grading efficacy for renal injury. These findings demonstrated the potential value of a non-invasively multi-parametric MRI approach for grading the renal injury preoperatively.

Multi-parametric MRI has been widely used to detect and evaluate kidney injury in different pathological conditions. The multiple quantitative MRI parameters were sensitive to detect the change of tissue microstructure during the pathophysiological processes. The combination of these MRI imaging biomarkers provides the possibility for identifying severity of kidney injury. The renal blood flow measured by ASL represents the renal perfusion [21], when the cortical and medullary Fp and Dp derived from IVIM represents microcirculatory perfusion, different from pure molecular diffusion D which is separated from ADC derived from monoexponential DWI [22]. The higher Mk values derived from DKI suggest more complex of tissue and are correlated with micro-structural heterogeneity [23–26]. BOLD MRI can assess renal oxygenation level and T2* value reduces in damaged kidney due to paramagnetic property of deoxyhenmoglobin [27, 28]. It has been reported that Md derived from DKI and Fp, D, T2* can be used to evaluate the severity of renal pathology and function in CKD patients [26, 27]. The T1 value measured by T1 mapping mainly depends on the amount of water molecules and may elevate while interstitial edema, inflammatory cell infiltration occur in tissue [29, 30]. Buchanan et al. combined T1 mapping, ASL and BOLD to assess acute kidney injury(AKI) and found that increased cortical and medullary T1, reduced cortical perfusion and shortened R2*, which suggested edema, inflammation of the renal parenchyma, hypovolaemia and renal hypoxia due to damage to the microvascular system [30].

In this study, medullary and cortical Dp and T2*, cortical Fp, ∆T1, cortical RBF, medullary T1 were selected out to predict grading the kidney injury. The medullary and cortical Dp and cortical Fp values are related to the liquid flow velocity and microcirculation blood volume, and cortical RBF reflect renal perfusion. This may suggest that the microvascular system is disordered among different extent of kidney injury during cold ischemia-reperfusion injury process. There may be several mechanisms resulting the changes. After renal cold ischemia and reperfusion, the renal interstitial edema and endothelial cell swelling results in renal capillary network damaged at early stage [31–33], then renal injury gradually recovered when some could return to normal levels. In addition, because vascular permeability increases, cellular structural is damaged and renal function reduces [34, 35]. This also can explain that the renal oxygenation state and amount of water molecules have a series of changes. However, the ADC, D, Md and MK were not filtered into the predicting model. This may suggest that although both water diffusion limited and microstructures complex after CIRI, the vascular and oxygen changes are more significant. Thus, this can explain IVIM and BOLD MRI technique had the higher AUC for pathology grading of renal injury. Cold ischemia-reperfusion injury is the result of a series of events, including hemodynamic changes, direct damage to cells and tissues, inflammatory cell infiltration, and obstruction of renal excreta. By combining multiple MRI parameters, we can effectively capture the key biological insights related to cellularity, water diffusion, blood oxygen metabolism, blood flow perfusion and water content. And MR parameters are relatively stable compared to creatinine and non-invasive. Artificial intelligence can integrate multiple sequence parameter features of MRI and merge these features into machine understandable codes for grading renal injury.

One of the significant problems of the classification research, the data imbalance must be considered. Therefore, necessary preprocessing is needed to prevent imbalance of models, which includes performing k-fold cross-validation, selecting relevant features, oversampling, undersampling (for example, synthetic minority oversampling technique, Tomek Links, et al.) [36, 37]. We tried to use 5-fold cross-validation on multiple machine learning models and observed the final test results. And we used the average accuracy, area under curve(AUC) and F1-score as the evaluation indicators. The results showed that the three models(multinomial logistic regression, random forest and support vector machine) had higher accuracy, F1 score and AUC. This indicates that each group has representativeness for each category.

Recently, Li et al. [38] showed that a logistic regression model by the machine learning approach with radiomic features derived from kidney ADC maps was able to distinguish CKD with healthy volunteers. Furthermore, Mo et al. developed a MRI texture-based machine learning model to evaluate renal function, which was based on the T2-weighted images for patients with diabetes [39]. In addition, Hara et al. created a random forest classifier model based on texture features of T1WI Dixon to evaluate renal dysfunction [40]. Currently, to the best of our knowledge, there are rare machine learning models been explored to predict the pathological grade for kidney injury based on the combination of ASL, BOLD, T1 mapping and DWI, IVIM, DKI MRI techniques. In our study, the dependent variable was an orderly classification variable, so we then used seven suitable machine learning classifiers and compared the efficiency of different classifiers for pathological grading. NN and KNN models had the lower AUC and accuracy than others, which may be associated with less input information and less samples. Random forest combines multiple decision trees for classification, randomly selects features and samples, and reduces the risk of overfitting [41]. SVM is a hyperplane supervised learning algorithm that only uses a portion of the support vectors to create a hyperplane. Our study showed that the accuracy, AUC and F1-score of multinomial logistic regression, RF and SVM were significantly higher than others models. Among them, multinomial logistic regression is easy to realize and understand classification decision based on probability value. Therefore, we can directly obtain the probability of predicting the degree of kidney injury quantitatively based on the optimal parameter values, and this model is robust. Both the density plots and radar plots for different techniques showed the stability of the models. Our findings may stimulate further research to determine whether multi-parametric MR-based machine learning model can early evaluate the extent of renal injury instead of biopsy and add prognostic value independent of clinical characteristics.

Nonetheless, there were several limitations in our study. First of all, we only evaluate single pathological state of CIRI model, while the numbers of rats were relatively small. Animal models can avoid complex clinical factors and simulate varying degrees of kidney injury under different pathological state. With more rats of renal injury under different injury mechanism, the performance of machine learning models for grading renal injury could be improved. Secondly, this was a single-center research, and lack of external validation. Our study was trying to explore a noninvasive method for preoperative prediction of the extent of kidney injury. Thus, prospective cohort researches with large samples, different disease states, different protocols and equipments from multicenter will be needed to promote the application in the further work. Thirdly, we construct the machine learning model only based on the parameter maps, more researches will be needed to identify whether T2WI as the routine sequence, or the texture features or radiomic features can be able to improve the accuracy for grading. Finally, more stable classification models combined with external validation for evaluating the degree of renal damage in patients will be need to explore in the future studies.

## Conclusions

We developed a machine learning model based on multi-parametric MRI to evaluate renal injury. This offers an alternative tool for grading the degree of renal injury noninvasively, based on the probability obtained directly from the machine learning model. In conclusion, the machine learning models based on multiple MRI biological markers will promote future studies of nephropathies in human to predict the severity of renal injury and add prognostic value independent of clinical characteristics.

### Electronic supplementary material

Below is the link to the electronic supplementary material.

Supplementary Material 1

## Data Availability

The datasets used or analyzed during the current study and machine learning model are available from the corresponding author on reasonable request.

## Abbreviations

ADC: Apparent diffusion coefficient
ASL: Arterial spin labeling
AUC: Area under curve
BOLD: Blood oxygen with blood oxygen level-dependent
BUN: Blood urea nitrogen
CIRI: Cold ischemia-reperfusion injury
D: Pure molecular diffusion
DKI: Diffusion kurtosis imaging
Dp: Pseudodiffusion coefficient
DWI: Diffusion weighted imaging
FOV: Field of view
IVIM: Intravoxel incoherent motion
KIM-1: Kidney injury molecule-1
KNN: K Nearest Neighbors
LASSO: The least absolute shrinkage and selection operator
Logistics: Multinomial logistic regression
Md: Mean diffusivity
Mk: Mean kurtosis
MRI: Magnetic resonance imaging
NGAL: Neutrophil gelatinase-associated lipocalin
NN: Neural network
RBF: Renal blood flow
RF: Random forest
SCr: Serum creatinine concentration
SVM: Support vector machine
TE: Echo time
TI: Inversion time
TR: Repetition time
