# Supplementary material for: Multi-parametric MRI-based machine learning model for prediction of pathological grade of renal injury in a rat kidney cold ischemia-reperfusion injury model
Source: BMC Med Imaging. 2024 Jul 26;24:188. doi: 10.1186/s12880-024-01320-6 (PMC11282691; doi:10.1186/s12880-024-01320-6)
Supplement: Supplementary file 1 — Supplementary Material 1 [file 12880_2024_1320_MOESM1_ESM.docx]

**Supplementary material**

**S1.Pathological scoring criteria**

| **Table S1. The pathological scoring criteria.** | | |
| --- | --- | --- |
|  | **The scoring criteria** | **Score** |
| **1** | The renal tubules significantly dilated, with the presence of tubular epithelial cell flattening | 1 point |
| **2** | Brush border damaged  Brush border loss | 1 point  2 points |
| **3** | Hyaline degeneration | 1 point |
| **4** | Interstitial edema | 1 point |
| **5** | If there are detached necrotic cells in the renal tubular lumen which have not formed tubular shape or fragment;  if there is tubular shape or fragment formed | 1 point  2 points |
| **6** | Nucleus pyknosis | 1 point |

**S2. Interobserver agreement**

| **Table S2. The ICC and 95% confidence interval of cortical and medullary MRI parameters between two observers.** | | |
| --- | --- | --- |
|  | **Cortex**  (95% CI) | **Medulla**  (95% CI) |
| **ADC（×10^−3^ mm^2^/s）** | 0.735（0.616-0.822） | 0.934（0.899-0.957） |
| **D（×10^−3^ mm^2^/s）** | 0.773（0.667-0.848） | 0.921（0.879-0.948） |
| **Dp（×10^−3^ mm^2^/s）** | 0.885（0.826-0.925） | 0.927（0.889-0.953） |
| **Fp（%）** | 0.893（0.838-0.930） | 0.833（0.752-0.890） |
| **T2*** | 0.772（0.666-0.847） | 0.888（0.831-0.927） |
| **Mk** | 0.827（0.742-0.885） | 0.780（0.677-0.853） |
| **Md（×10^−3^ mm^2^/s）** | 0.946（0.917-0.965） | 0.807（0.714-0.872） |
| **T1（×10^2^ ms）** | 0.781（0.678-0.854） | 0.783（0.681-0.855） |
| **RBF（ml·100g^-1^·min^-1^）** | 0.992（0.988-0.995） | - |
| ICC: Intraclass correlation, CI: confidence interval, ADC:apparent diffusion coefficient, D: pure molecular diffusion, Dp: pseudodiffusion coefficient, Fp: perfusion fraction, Mk: mean kurtosis, Md: mean diffusivity, RBF:renal blood flow. | | |

**S3. Predictive Model Construction for grading renal injury**

| **Table S3. The accuracy, AUC and F1 score of each classifier for predicting pathology grade.** | | | |
| --- | --- | --- | --- |
| **Classifier** | **Accuracy** | **AUC** | **F1 score** |
| **Logistics** | 0.83 | 0.95 | 0.74 |
| **RF** | 0.85 | 0.93 | 0.83 |
| **SVM** | 0.81 | 0.89 | 0.73 |
| **Bayes** | 0.74 | 0.89 | 0.72 |
| **Decision Tree** | 0.78 | 0.81 | 0.72 |
| **KNN** | 0.66 | 0.78 | 0.56 |
| **NN** | 0.65 | 0.75 | 0.64 |
| AUC: area under curve, Logistics: multinomial logistic regression, RF: random forest, SVM: support vector machine, KNN: K nearest neighbors, NN: neural network. | | | |
